# EXPLORING THE POTENTIAL OF CHATGPT FOR ASSESSING DELIRIUM SYMPTOMS IN OLDER ADULTS WITH COGNITIVE IMPAIRMENT

**DOI:** 10.1093/geroni/igad104.0990

**Published:** 2023-12-21

**Authors:** Yong Kyung Choi, Shih-Yin Lin

**Affiliations:** University of Pittsburgh, Pittsburgh, Pennsylvania, United States; NYU Rory Meyers College of Nursing, New York City, New York, United States

## Abstract

Delirium is a common and serious geriatric syndrome, especially in older adults with cognitive impairment. Accurate caregiver assessment and reporting of delirium in community-dwelling older adults can help healthcare providers diagnose and treat delirium more effectively. Yet, caregivers often lack confidence in their ability to accurately assess and report delirium. An automated method to detect the presence and symptoms of delirium could potentially boost caregiver confidence and assist with more accurate delirium assessment and reporting. This study explored the use of ChatGPT, a generative AI based on the Large Language Model (LLM), to detect the presence and symptoms of delirium. We trained ChatGPT using a series of prompts specifically designed to enhance its understanding of the domain knowledge and the scoring mechanism of a caregiver-centered, seven-item delirium assessment, the Sour Seven. The trained ChatGPT model was asked to identify and score various delirium symptoms in five previously validated geriatric case vignettes using the Sour Seven scale. Each case was repeated three times to test for reliability. The ChatGPT assessment results were compared against those of human experts. The preliminary analysis showed that items 3, 6 and 7 were scored correctly in all cases, but mixed results were found on items 1, 2, 4, and 5, mostly because the AI applied a single symptom statement to multiple items. Overall, the results indicate that generative AI has the potential to accurately capture and represent complex delirium symptoms in natural language, which could assist caregivers in accurately identifying and reporting delirium symptoms.

